# Heat Exchange in Adiabatic Calorimeters

**DOI:** 10.6028/jres.067A.035

**Published:** 1963-08-01

**Authors:** E. D. West

## Abstract

Heat flow in adiabatic calorimeters of various shapes and materials is described in terms of linear partial differential equations. From these equations it is deduced that in the intermittent heating method the heat exchange between the calorimeter and the adiabatic shield due to transients at the beginning and end of the heating period can be made to cancel. The remaining heat exchange is the same for intermittent or continuous heating methods and can be treated as the sum of effects due to gradients set up by heat flow (1) from the shield to the environment and (2) from the shield and calorimeter heaters to raise the temperatures of the shield and calorimeter, respectively. The first effect can be accounted for by measurements during fore and after periods in intermittent calorimetry and by varying the heating rate in continuous calorimetry. Under certain conditions the second effect can be accounted for by measurements with the empty calorimeter. Variation in heating rate fails as a test for the magnitude of the second effect.

## 1. Introduction

Standard materials for heat capacity measurements have now been used widely enough to show that different observers obtain data with systematic differences not ascribable to variations in samples. Well-established techniques for measuring electrical energy and temperature differences are accurate enough that the most likely source of the differences is in accounting for heat exchange between the calorimeter and its surroundings. A review of the ideas on which such calorimetry is commonly based therefore seems to be in order.

Heat flow in calorimeters is commonly analyzed using Newton’s Cooling Law. The calorimeter and its surroundings are treated as isothermal bodies and the heat transfer between them as proportional to the differences in their temperatures. The constant of proportionality is the integral for all the radiation and conduction processes, which can properly be lumped together only if the same temperature difference applies to all of them. To satisfy this model, stress is laid on making the surfaces isothermal. The model is usually abandoned at this point to consider the temperature gradients. The attempt is often made to locate temperature sensing elements to average the temperature over the surface. The consideration of gradients also requires consideration of the variation in heat transfer coefficients over the surface, and some calorimeters use separate temperature measurements to account for surface radiation and for lead conduction. The accuracy of calorimeters designed using this model hinges on the intuition and experience of the designer in going beyond the model to allow for the effect of temperature gradients. A good example of how this method can lead to wrong conclusions is the use of the heating rate to demonstrate that the surface is adequately isothermal.

In principle, an adiabatic calorimeter is one in which heat is confined to the calorimeter usually by surrounding it with an adiabatic shield maintained at the temperature of the calorimeter. In practice, temperature gradients in the calorimeter and shield cause a net heat exchange during the experiment. The heat exchange during an experiment is commonly accounted for by the use of measurements at other times. In the intermittent heating method, for example, heat corrections to the energy input during the experiment depend on drift rates and heat transfer coefficients measured with no energy input and on the heat capacity of the empty calorimeter measured at a more remote time. The assumptions and approximations implied in such corrections and the experimental conditions necessary to insure their validity need to be reviewed for possible sources of the discrepancies in heat capacity data.

Variation of the heating rate is a time-honored test for adiabatic calorimeters [1–11, 22].^1^ It recognizes that there are small temperature differences on the surface of the calorimeter and adiabatic shield during the heating period. A lower heating rate will decrease the temperature differences and the corresponding rate of heat exchange between the calorimeter and shield. If the same heat capacity is observed at very different heating rates, it is sometimes argued that the difference in heat exchange and hence the heat exchange itself must be negligible. This line of reasoning has been challenged [12] on the ground that it fails to consider the time required for an experiment. Since heat capacity is a function of temperature, comparisons must be made over the same temperature interval. Except for transient effects, the surface temperature differences and the corresponding rate of heat exchange are approximately proportional to the heating rate, but the time required to heat through a given temperature interval is inversely proportional to the heating rate. Since the net heat exchange is the product of the rate of heat exchange and the time, it is practically the same for all heating rates. This unknown net heat exchange must be accounted for by making it the same in the experiments with the full and the empty calorimeter.

Starting with the partial differential equations describing the heat flow in a calorimetric apparatus, the mathematical analysis in the present paper will show that the transient effects, which weaken the qualitative argument [12], can be made to cancel for each experiment. The heating rate test for isothermal surfaces must then fail and the difference in the unknown heat exchange between measurements with the full and the empty calorimeter must be admitted as a cause of systematic deviations.

## 2. Statement of the Problem

### 2.1. Qualitative Description

The problem is to describe mathematically the heat flow in a calorimetric apparatus and to see what consequences can be deduced. The experiment will first be described qualitatively. A calorimeter is surrounded by an imperfect adiabatic shield. The temperature difference between the two is sensed at one or more points, usually with a thermopile. The temperature of the shield as observed at these points is controlled to be equal to that of the calorimeter. The treatment includes any supports, leads, and gas between the physical shield and the environment, which is taken to be at constant temperature.

The temperature of the calorimeter is observed during the initial equilibrium period until its rate of change becomes very small and constant within the limits of observation. The initial temperature of the experiment is then taken. Constant power is then supplied to the calorimeter for a measured time. The power is turned off and, after the rate of change of the calorimeter temperature again becomes constant, the final temperature is taken.

Usually the rate of change or “drift” of the calorimeter temperature in the equilibration periods is not zero, indicating that the temperature of the adiabatic shield is not perfectly matched to that of the calorimeter even when the controlled temperature difference is zero. The heat exchange indicated by this temperature change will be referred to subsequently as the “zero heat leak.”

Whenever heat is supplied to the calorimeter or the adiabatic shield, it must flow from the heater to a surface where it is lost to other surfaces or to some volume where it raises the temperature. This heat sets up gradients throughout the body which have components along the surface. Since the temperature of the calorimeter and shield can be observed at only a relatively few points, it follows that the observed temperature differs from the average over the surface. Consequently, there is heat exchange between the calorimeter and the shield whenever power is supplied to *either* of them, even though the controlled temperature difference is zero. It is possible and often practicable to distribute heaters and thermocouples to make this heat exchange small. It does not appear possible to satisfy exactly the requirements that a shield heater, for example, should be distributed so as to generate at each point at equilibrium the heat lost from that point to the environment and that the same distribution should supply both heat lost and heat required to raise the shield temperature during the heating period without gradients along the surface. Such an exact distribution will be even more difficult if it must take into account the variation in heat capacity of the components when the apparatus is used over a considerable temperature range.

It is also apparent from working with these calorimeters that some time is required after turning on the heater before all parts begin to heat at the same rate and that some time is required after turning off the calorimeter for the temperature to come to equilibrium. The mathematical treatment must take these transients into account.

### 2.2. The Differential Equations

The method of treating the problem is (1) to set up the differential equations and boundary conditions for heat flow in the calorimeter and the adiabatic shield, making a minimum number of assumptions; (2) to use the principle of superposition, which states that the sum of solutions to a linear differential equation is also a solution, in order to show the causes of the temperature gradients; (3) to indicate the nature of the solutions and show from them how the heat loss depends on the heating rate and other factors.

Throughout the discussion it should be kept in mind that we are analyzing the apparatus for a heat leak correction which is a small part of the total heat added in an experiment. We can therefore use approximations which affect the heat leak by only a small percentage of itself. To obtain linear equations, the approximations are made that the heat capacities per unit volume, the heat transfer coefficients, and the thermal conductivities of all materials in the calorimetric apparatus are independent of temperature over the small temperature interval of the experiment. These approximations are implied in most calorimetry, in which the temperature rise during the reaction or heating period is corrected for heat leak on the basis of observations in fore and after periods. During phase transitions in the sample, where two phases are present in changing amounts, this approximation may not apply. A proposal for overcoming this difficulty is discussed in sec. 6.2. Heat transfer by convection is assumed negligible.

The differential equation for heat flow by conduction and its solution are treated by several authors [13, 14, 15]. The method followed here of substituting a sum of solutions is discussed in some detail by Boley and Weiner [14].

Temperature and Heating Rate Symbols:

*T*: =the temperature distribution as a function of time and position. The scale is arbitrary, but it can be thought of as the Kelvin temperature.
*T*_0_: =the observed initial temperature of the thermometer in the calorimeter at the time the heater power is turned on.
*T_j_*: =final observed calorimeter temperature, corrected for zero heat leak.
*T*_0_*_e_*: =constant temperature of the environment, excluding supports, leads, and gas which are at different temperatures due to conduction from shield.
*L*: =the temperature distribution due to heat exchange between the shield and its environment, which causes the “zero heat leak,” sec. 2.1.
*θ*: =the temperature distribution due to the quasi-steady state set up by constant power in the calorimeter heater.
*τ* and τ^*^: =the initial and final transient temperature distributions.
*β*_L_: =rate of change of temperature due to heat leak.
*β_θ_*: =rate of change of temperature due to constant power in the calorimeter heater.

Subscripts:

*i, j*, *k*: differentiate the various regions of the calorimeter and shield under discussion.
*w*: designates the heater wire regions in which heat is generated.
*c*, *s, e*: refer to calorimeter, shield and environment, respectively.
*con*: refers to the particular position at which a control temperature is taken.

Other Symbols:

*P*: =power. *P_c_*= constant for a given experiment; *P_s_* contains transient terms.
*Q*: =heat.
*C*: =heat capacity per unit *volume*.
*h*: =radiation heat transfer coefficient (in dimensions power ÷ [area]^2^).
*K*: = thermal conductivity.
*t*: =time. (The heater is turned on at *t*=0, off at *t=t_f_*)
*A*: =area of a region of the calorimeter or shield.
*A_s_*: =sum of areas of shield regions which exchange heat with calorimeter and environment.
*V*: = volume.
*σ*: =Stefan-Boltzmann radiation constant.
*α*: = absorptivity.
*ϵ*: =emissivity.
*δ_iw_*: = l when *i=w*, 0 when *i≠w*. (Kronecker delta. Makes power term zero except in heater wire.)
  $$\nabla^{2}T=\frac{\partial^{2}T}{\partial x^{2}}+\frac{\partial^{2}T}{\partial y^{2}}+\frac{\partial^{2}T}{\partial z^{2}}$$
$\frac{\partial }{\partial n_{i}}$: =the partial derivative in the direction outward from and perpendicular to the surface of the *i*th region.

Because calorimeters are made up of a variety of materials, the analysis must treat them as composed of a number of homogeneous regions. The discussion will therefore treat a general *i*th region of the calorimeter or shield which is in thermal contact with one or more *j*th regions and may radiate to one or more *k*th. regions if the *j*th region is transparent.

The experiment to be described is one with a constant-temperature environment (e.g., an ice bath) and with a zero heat leak which is observed to be the same before and after the heating period. This condition is likely to be observed when the heat leak is small and the fractional change in the temperature difference between the shield and the environment is small. The differential equations and the various boundary and initial conditions are shown in table 1. The overall temperature distribution *T* satisfies the equations in column 11. The equation for heat conduction, II–A, is discussed by Carslaw and Jaeger [13] and by Boley and Weiner [14]. The equation in this form uses the approximation that the thermal conductivity is constant. The terms in *P_c_* and *P_s_* in equations II–A allow for power generation in the regions of the heater wires and are zero outside those regions. In the calorimeter heater, *P_c_* is independent of time during the heating period and zero otherwise. It is assumed that the power is developed uniformly over the volume of the wire so that the power per unit volume is just the total power divided by the total volume of the wire. The shield power *P_s_* is not constant because it is affected by the requirement that the controlled temperature difference between the shield and calorimeter be zero. The expression for the shield power is therefore derived from the requirement that energy is conserved and consists of the power used to raise the shield temperature plus the net power lost to the calorimeter and to the environment.

The general boundary condition, equation II–B, includes conduction from the *i*th region to the *j*th region. If the *j*th region is transparent, as in a space filled with a gas, the equation allows for radiation through that region to one or more *k*th regions. The radiation term is integrated over all *k*th regions, including the *i*th region. If the *i*th region is surrounded by opaque *j*th regions, the radiation heat transfer coefficient *h_ik_* is zero; if the *j*th region is evacuated, the thermal conductivity *K_j_* is zero and the continuity condition *T_i_* = *T_j_* is not required. This equation applies to all regions of the calorimeter and shield and governs the heat transfer between them (cf., sec. 3.2). The equation takes into account the geometric variation of the temperature, the thermal conductivity and the radiation coefficients; i.e., Newton’s law of cooling is not assumed to govern heat transfer between the calorimeter and the adiabatic shield. The radiation heat transfer coefficient *h_ik_* is derived from the Stefan-Boltzmann equation 2 [15, Vol. I].

Where leads, supports, or gas are in thermal contact with the constant-temperature environment, the boundary condition at the surface of contact is simply

$$T_{is}=T_{0e}.$$ (1)

The initial condition at *t*=0, II–C, allows for the temperature distributions due to the zero heat leak in the various regions of the calorimeter and shield just before the power is turned on in the calorimeter heater.

To facilitate the analysis, the experiment is considered in two parts—the heating period and the final equilibration period. A second “initial” condition, II–D, is therefore included for the time *t_f_* when the calorimeter heater is turned off. This temperature distribution must include (1) the *θ_i_* which are still present at time *t_f_* due to power required to raise the temperature of the calorimeter and shield; (2) the general increase in temperature due to rates of change of the calorimeter temperature *β_L_* and *β_θ_* due respectively to heat exchange with the environment and to constant power during the heating period; (3) the net contribution of the initial transient which, at *t_f_*, is merely a small constant *τ*_∞_ added to the temperature at each point in the calorimeter and shield.

### 2.3. Temperature Distribution Due to Heat Leak, Constant Power, and a Transient

In order to assess the effects of different surface temperature distribution during the heating and equilibration periods, it is desirable to consider the overall temperature distribution *T_i_* as a sum due partly to heat exchange with the environment and partly to power flowing in the calorimeter and the shield to raise their temperatures. With the approximation that the thermal conductivity, the heat capacity per unit volume and the heat transfer coefficients are independent of temperature over the small temperature rise of the experiment, the equations in column II, table 1, are linear in *T* and its derivatives. The *T_i_* may therefore be written as a sum of solutions [14, pp. 201 and 231]. For the calorimeter during the heating period

$$T_{ic}=L_{ic}+\beta_{L}t+\theta_{ic}+\beta_{\theta }t+\tau_{ic}+T_{0}.$$ (2)

The temperature where the control thermocouple is attached to the shield is controlled to equal the temperature *T_conc_* where the thermocouple is attached to the calorimeter. The temperatures in the shield are measured relative to this controlled temperature so that *T_is_* is given for the heating period by the sum

$$T_{is}=L_{is}+L_{conc}+\beta_{L}t+\theta_{is}+\theta_{conc}+\beta_{\theta }t+\tau_{is}+\tau_{conc}+T_{0}.$$ (3)

The boundary and initial conditions III–B, IV–B, V–B, table 1 are not quite complete as shown. Where they involve *T_i_–T_k_* and derivatives with respect to the normal, the terms in *t*, *T*_0_ and control temperatures do not appear when both the *i*th and *k*th regions are in the calorimeter, the shield, or the environment. But, for heat transfer between the calorimeter and shield or between the shield and environment, these terms must be retained, as in eqs (5) and (6). (See note, table 1.)

When the operations indicated in column II are performed on these sums the various terms can be separated as in columns III, IV, and V. The three sets of equations can be associated respectively with the observed zero heat leak which exists throughout the experiment, constant power in the calorimeter during the heating period, and transients allowing for the transitions between equilibration and heating periods.

The term *θ_i_* is the temperature distribution for the quasi-steady state [14, p. 201] due to the constant power input in the calorimeter during the heating period. It satisfies the equations in column IV, table 1, and is a function of geometric variables only. Where the boundary conditions involve the temperature of the environment, *θ_k_* is zero for radiation and *θ_j_* is zero for conduction, since the environment temperature does not contain these terms (cf., eqs (1) and (6)). It can be verified by differentiation and substitution in the equations that, if *θ_ic_* is the temperature distribution and *β_θ_* the heating rate for a power *P_c_, mθ_ic_* is the temperature distribution and *mβ_θ_* the heating rate for *mP_c_*; i.e., the quasi-steady state temperature gradient and the heating rate are directly proportional to the calorimeter power.

To allow for the transients in going from the equilibration conditions to heating conditions and the reverse, the transient terms *τ_i_* and $\tau_{i}^{*}$ are included. These terms make the sums in eqs (2) and (3) satisfy the initial conditions II–C and II–D when the calorimeter heater power is turned on and off. Where the boundary conditions involve the temperature of the environment, *τ_k_* is zero for radiation and *τ_j_* is zero for conduction since the environment temperature does not contain these terms (cf., eqs (1) and (6)). The two transients, one, *τ*, when the power is turned on and one, τ*, when it is turned off, satisfy identical sets of equations, except for their initial conditions, V–C and V–D, which are equal but of opposite sign. Considering the final transient τ* a function of (*t−t_f_*), it is apparent from substituting in the equations of column V that, for equal magnitudes *a* of (*t−t_f_*) and *t*,

$$-\tau^{*}(a)=\tau (a).$$ (4)

The term *β_L_* is the slow change of the calorimeter temperature due to the zero heat leak. It is equal to the net power transferred between the calorimeter and the shield during the equilibration periods divided by the heat capacity of the calorimeter. It may be either positive or negative depending largely on the relative locations of the heater and control thermocouple on the adiabatic shield. The term *β_θ_* is the rate of rise of the calorimeter temperature due to constant power in the calorimeter heater. It is equal to the sum of this power and the small power flowing from the shield due to surface temperature distributions *θ* set up during the heating period divided by the heat capacity of the calorimeter.

The term *L_i_* is the “zero heat leak” temperature distribution, due to gradients produced in the shield by heat transfer to the environment. As a consequence of eq (1), eq III–B, table 1 takes a slightly different form when the regions *j* or *k* are the environment. For radiation to the environment, the relation is

$$\begin{array}{l} K_{is}\frac{\partial L_{is}}{\partial n_{is}}=K_{js}\frac{\partial L_{js}}{\partial n_{is}}+\int_{A_{k}}h_{ik}(L_{ks}-L_{is})dA\\ \;+\int_{A_{e}}h_{ie}[T_{0e}-L_{is}-L_{conc}-T_{0}-(\beta_{L}+\beta_{\theta })t]dA \end{array}$$ (5)

where integration over the *k* regions does not include the environment. For conduction, where leads, supports, and gas are in thermal contact with the environment,^3^ the boundary conditions reduce to the constant temperature on the surface:

$$L_{is}+L_{conc}+\beta_{\theta }t+\beta_{L}t+T_{0}=T_{0e}.$$ (6)

In eqs (5) and (6), the term *β_θ_t*, which has elsewhere been associated with constant power input to the calorimeter, is included because it increases the temperature difference for radiation between the shield and the environment and the value of *L_is_* at conduction boundaries. These quantities cause the heat flow from the shield, which sets up the zero heat leak temperature distribution.

To satisfy the observed condition that the zero heat leak is the same in the initial and final equilibration periods, the effect of the increase in temperature on *∂L_is_/∂n_is_* must be negligible. The effect of (*β_L_+β_θ_*)*t* must therefore be undetectably small and these terms are neglected in column III, table 1. To this approximation, the *L_i_* are independent of time.

## 3. Interpretations of the Equations

### 3.1. The Thermal Transients

**Figure 1:**
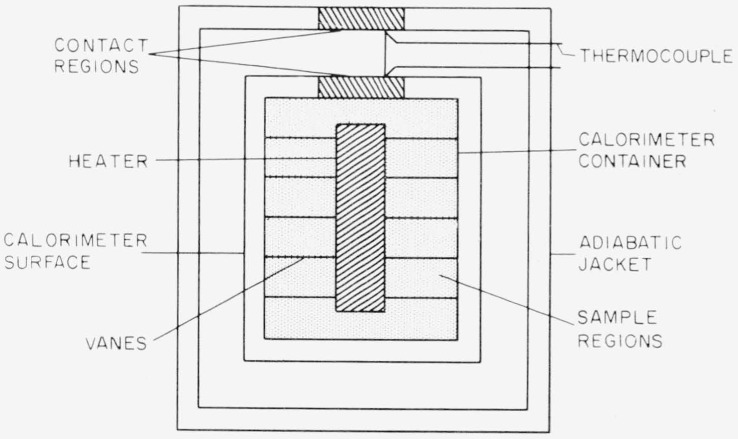
Idealized cross section of a calorimeter.

**Figure 2:**
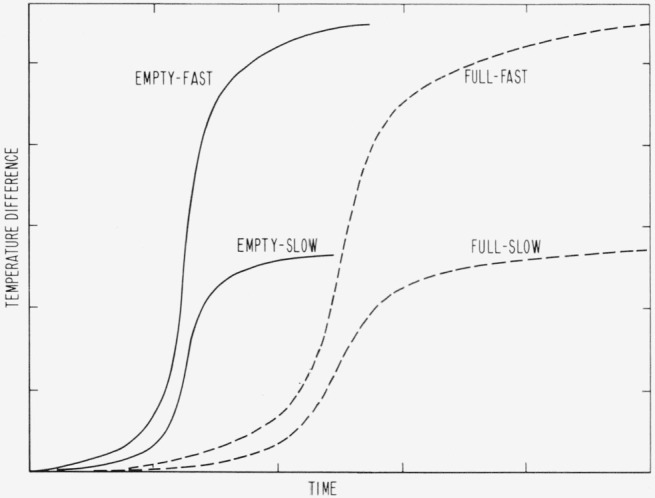
Schematic of temperature difference between two points on the surface of the calorimeter as a function of time for different heating rates and loading conditions.

Good practice in adiabatic calorimetry is to make measurements with the calorimeter full and empty (or with large and small samples) reproducing the gradients on the surfaces of the calorimeter and shield in order to allow for the unknown heat transfer during the heating period. But placing a sample in the calorimeter alters the time required for heat to reach the surface, so that the transients for the full calorimeter differ from those for the empty. This effect is more easily visualized by reference to an example. Figure 1 is an idealized cross section through a calorimeter consisting of coaxial cylinders. For simplicity, it is assumed that heat transfer is by solid conduction only. Heat then flows from the heater along the vanes to the container wall, through the contact region to the calorimeter surface. If the temperature for control is measured at one point on the surface of the calorimeter, then, during the heating period, another point on the surface will be at a somewhat different temperature. During the equilibration period, the temperature difference between these two points will be very small. During the transient period, the temperature difference goes from one condition to the other, but the *time* required must depend on the thermal conductivity and heat capacity of the sample. During the initial transient, the curve for the empty calorimeter might resemble the empty-slow curve (fig. 2), for some heating rate *β_θ_* If the heating rate is 2*β_θ_*, the time and geometric dependence are unchanged and the amplitude is doubled, as in the empty-fast curve.

If the calorimeter is now filled with a material of high heat capacity and low thermal conductivity, the time for heat to reach the surface will be greatly increased. With the assumption that all heat reaches the surfaces by way of the contact regions, the temperature difference between two points will ultimately come to the same values for the same heating rate. For a heating rate of *β_θ_* in the full calorimeter, the full-slow curve might be obtained, and, for 2*β_θ_*, the full-fast curve. Evidently the curves for the empty calorimeter do not compensate for the curves for the full calorimeter. That no error results from this difference is due to compensation of the final transient for the initial transient in each experiment. This compensation will be shown in the next section.

From eq V–B an explicit equation for *τ_is_*, like that for *L_is_* (eq (5)), can be written to show that the transients depend on conduction paths to the constant-temperature environment. If the calorimeter is to reach equilibrium quickly, these conduction paths must be short or of low heat capacity especially for poor thermal conductors.

### 3.2. The Unknown Heat Loss During the Heating Period

It is now possible to draw conclusions regarding the total heat exchange between the calorimeter and the shield during an experiment. Using the definition of thermal conductivity and integrating over the entire calorimeter surface and the time of the experiment, including the final equilibration period, the total heat loss *Q* from the calorimeter is

$$Q=\int_{0}^{t_{r}}\int_{A_{c}}K_{ic}\frac{\partial (\theta_{ic}+L_{ic}+\tau_{ic})}{\partial n_{ic}}dAdt+\int_{t_{f}}^{t_{r}}\int_{A_{c}}K_{ic}\frac{\partial \tau_{ic}^{*}}{\partial n_{ic}}dAdt.$$ (7)

Observations of the zero heat leak in the final equilibration period are continued until the time *t_r_* at which the transients are no longer observable.

The experiment is arranged so that *τ* and τ* become constant and equal to *τ*_∞_ at a time *t*_1_*<t_r_*. It was shown in sec. 2.3 that, for equal magnitudes *a* of *t* and *t−t_f_, τ*(*a*) = −τ*(*a*) Their integrals up to *t*_1_ are therefore equal and opposite and make no net contribution to *Q*.

The quantities remaining in eq (7) are independent of time, so that the time integration may be performed to obtain

$$Q=t_{f}\int_{A_{c}}K_{ic}\frac{\partial (\theta_{ic}+\tau_{\infty })}{\partial n_{ic}}dA+t_{r}\int_{A_{c}}K_{ic}\frac{\partial L_{ic}}{\partial n_{ic}}dA.$$ (8)

The last integral is simply the zero heat leak multiplied by the time elapsed between the initial and the final temperature readings. This integral is evaluated from determinations of *Q* over a timed interval during the equilibration periods. The remaining unknown heat leak is that due to gradients set up during the heating period.

The heat exchange due to *τ*_∞_ is included for generality, but its practical effect is negligible. It is the net effect of the initial transient on the calorimeter temperature, a small constant added to the temperature at all points in the calorimeter. Since the shield control will change the shield temperature to allow for *τ*_∞_, the direct effect on the calorimeter is zero. The slight change of *τ*_∞_ in the shield-to-environment temperature difference produces a much smaller effect than that due to the temperature rise during the experiment.

An estimate can be made of the maximum effect of *τ*_∞_, assuming that all of the nearly 1 percent scatter in heat capacity data for Al_2_O_3_ obtained by various experimenters is caused by variation in heat exchange due to the *θ_i_* during the heating period. This scatter corresponds to 1 percent of the temperature rise *β_θ_t_f_*. The effect of *τ*_∞_ is related to that of *θ_i_* by the ratio of its “time constant” to *t_f_*, the length of the heating period. The zero heat leak would have to be quite large for 1 percent of it to be important.

Since the first term contains *t_f_*, the amount of heat exchange depends on the length of the heating period. It might appear to be possible to evaluate the integral by observing the apparent heat capacities as a function of *t_f_*, corrected for the zero heat leak. The fallacy in this reasoning is easily demonstrated. The time *t_f_* required to heat through the temperature range *T_f_*−*T*_0_ (corrected for zero heat leak) over which comparison is made is given by the equation

$$t_{f}=\frac{T_{f}-T_{0}}{\beta_{\theta }}.$$ (9)

Combining (8) with (9), the unknown part of the heat exchange is seen to be independent of the heating rate since increasing the power by a factor *m* increases *β_θ_*, *θ*, and *τ* by the same factor. The unknown heat exchange is proportional to the temperature rise in an experiment, independent of the time of the heating period. It cannot be separated from the true heat capacity by changing the heating rate.

This conclusion contravenes the common practice of varying the heating rate to demonstrate that temperature differences on the surfaces of the calorimeter and shield are negligible, as has sometimes been asserted [7, 22]. The conclusion is borne out by measurements in this laboratory [12]. Observed heat capacities at one-half the usual heating rate agreed with the averages of the other data to 1 part in 25,000, although the temperature differences on both the calorimeter and shield surfaces were calculated and/or observed to be about one-tenth of a degree.

### 3.3. The Zero Heat Leak and Parasitic EMF’s

The zero heat leak, which is due to temperature distributions set up by heat lost from the adiabatic shield, is present throughout the experiment. The present analysis is based on the observation that the rate of change of the calorimeter temperature is the same in the initial and final equilibration periods. It is conceivable that such an observation might be due to compensation of increased heat transfer and increased heat capacity of the calorimeter, but the experiment provides a direct check on the latter. For a small correction, as the zero heat leak should be, the ordinary variations in heat capacity with temperature can be ignored. The correction for the observed constant rate is simply the rate times the time elapsed between the initial and final temperature readings, and, for approximately constant heat capacity, the correction is equally well applied to the temperature rise or, after multiplication by the heat capacity, to the energy term.

There is nothing in the equations to indicate the sign of the zero heat leak, and, in most apparatus, heat probably flows from the calorimeter at some points and to it at others. The net effect depends on the relative locations of the shield heater and the control couple if there is no parasitic emf in the control circuit.

A constant parasitic emf in a lead of the control thermocouple can be taken into account by adding a corresponding constant temperature difference to *L_conc_* (eqs (5) and (6), and III, table 1). Whatever changes the parasitic emf induces in the *L_i_* persist throughout the experiment, and the proper correction is made based on observations during the equilibration periods.

The same reasoning applies to an intentional constant offset in the control apparatus. Accordingly, no error is introduced when the control point is deliberately shifted to make the observed temperature change during the equilibration period zero. This technique can be used to make temperature measurement easier (see sec. 6.1). One should not be deceived that gradients on the calorimeter are eliminated by this technique; it is merely a shift of the distribution on the shield relative to the distribution on the calorimeter to balance the small heat flow to the calorimeter against the heat flow from it.

## 4. Calorimeters With Temperature-Controlled Environment

When it is desirable to eliminate the effect of *β_θ_t* on the zero heat leak or when *β*_L_ is large enough to cause difficulty with the temperature measurement, it is feasible to reduce them by providing a heated environment controlled about a degree below the temperature of the shield [5, 12, 16, 17]. This technique is especially useful at higher temperatures where the heat transfer coefficients are large.

Since the environment has a dynamic temperature variation in this case, the effect of the temperature gradients from the various sources must be considered and allowance made for various environment materials. The environment temperature will now be controlled relative to some point on the shields where the temperature is *T_cons_*, and will have temperature distributions due to heating up and due to loss to the environment through the thermal insulation. The temperature of the *i*th region of the environment is given by the equation

$$T_{ie}=T_{0}-\Delta T+L_{ie}+L_{cons}+\theta_{ie}+\theta_{cons}+\tau_{ie}+\tau_{cons}+(\beta_{L}+\beta_{\theta })t,$$ (10)

where Δ*T* is the controlled difference between the points on the shield and on the environment where the thermocouple junctions are located. This expression for *T_ie_* combined with equations II–B, table 1, gives the heat flow to the environment from each point on the shield:

$$\begin{array}{l} K_{is}\frac{\partial T_{is}}{\partial n_{is}}=K_{je}\frac{\partial (L_{je}+\theta_{je}+\tau_{je})}{\partial n_{is}}\\ \;+\int_{A}h_{ik}(L_{ke}+L_{cons}+\theta_{ke}+\theta_{cons}+\tau_{ke}+\tau_{cons}-L_{is}\\ \;-L_{conc}-\theta_{is}-\theta_{conc}-\tau_{is}-\tau_{conc}-\Delta T)dA_{e} \end{array}$$ (11)

where gas regions are now arbitrarily part of the environment. On opaque conduction boundaries, *T_is_=L_je_+θ_je_+τ_je_*. This equation does not now contain explicitly the *β_θ_t* term which caused the variation in the zero heat leak, but the corresponding expression for the heat loss from the controlled environment will contain *β_θ_t*. Consequently, the validity of the approximation that increasing the environment temperature by *β_θ_t* has a negligible effect on the zero heat leak must still be verified by observation.

When eq (11) is separated into a sum it becomes apparent that all the transients depend on the transients of the environment. Whether these *τ_ie_* have a negligible effect on the shield temperature distribution is subject to observation. A large environment insulated with a considerable mass of material may so extend the equilibrium time that observations of the apparent zero heat leak will contain contributions from the transients. It is not necessary to wait for the environment transients to die out if the zero heat leak is negligibly affected by the small variations in the environment temperature, an effect observed with an adiabatic calorimeter operated up to 400 °C in this laboratory [12]. In constant heating calorimeters (sec. 5) the temperature of the controlled environment can be made equal to that of the adiabatic shield, so that, except for stray thermoelectric emfs, the zero heat leak depends only on the small effect on the shield of the gradients in the environment. This effect has been observed to be negligible in a low-temperature calorimeter [11].

Two experimental checks on the environment are available. The obvious one is to look for the variation in a zero heat leak after the calorimeter power is turned off. If the leak is constant, either the *τ_ie_* are short or have negligible effect on the shield. Another test is to increase the controlled temperature difference between the shield and environment by an amount larger than the most unfavorable estimate of the quasi-steady state temperature distribution in the environment, *θ_ie_*. If the zero heat leak is not affected by this change, it should be independent of the transients in the environment because their maximum value is just *θ_ie_*.

## 5. Continuous Heating Calorimeters

In the continuous heating method, data are taken without an equilibration period. Measurements are begun after the transient terms, column V, table 1, have become negligible. The observed heat capacity *C_obs_* is related to the power input to the calorimeter by the equation

$$C_{\text{obs}}\frac{dT}{dt}=P_{c}+\int_{A_{c}}K_{ic}\left(\frac{\partial \theta_{ic}}{\partial n_{ic}}+\frac{\partial L_{ic}}{\partial n_{ic}}\right)dA.$$ (12)

Only two terms appear under the integral because the normal derivatives of the other terms (eq (2)) are zero. Integration is over the entire calorimeter surface *A_c_*. This equation states that the power going to raise the temperature of the calorimeter is diminished by losses due to (1) gradients set up by power supplied from the shield to the environment and (2) gradients set up by power supplied to raise the calorimeter and shield temperatures. It was shown in sec. 3.2 that the power term and the *θ_i_* integral in eq (12) are directly proportional to the heating rate. If measurements of *C_obs_* are made at two heating rates and plotted against heating rate, the intercept for zero heating rate will give the heat capacity corrected for the zero heat leak. The intercept method of determining the zero heat leak assumes that the various parameters affecting the zero heat leak are constant between series of experiments. Thus, if there is a gradient across an inhomogeneity in a thermocouple lead wire producing an offset in the shield control, the assumption is that the same gradient is present in both series of measurements. The determination of the zero heat leak by initial and final equilibration experiments assumes the constancy of such effects over a shorter period.

Theoretically, then, there is no basis for choice between the two methods of constant heating rate and alternating equilibration with heating. The choice of method depends on practical matters, such as control problems, the nature of the sample, and the length of time conditions can be expected to remain sufficiently constant to yield significant zero heat leak data.

## 6. Design and Testing of Adiabatic Calorimeters

Several conclusions can be drawn regarding testing and construction of calorimeters, some of which support practices in design and testing arrived at by less general methods.

### 6.1. Temperature Measurement Error

Because of temperature variations due to the zero heat leak, the thermometer for making absolute temperature measurements in the calorimeter will in general not be so located as to obtain the average temperature. The error may be appreciable where the absolute temperature is important, as in the determination of the temperature at which phase changes occur. Even if the observed calorimeter temperature is constant, the absolute temperature may still be in error due to heat flow into one part of the calorimeter and out from another (sec. 3.3). In heat capacity measurements, if the heat leak temperature distributions *L_i_* are constant throughout the experiment, the region in which the temperature is measured will bear the same relationship to the average over the calorimeter before and after the heating period. The errors cancel for each individual experiment. It follows that the location of the sensing element in the calorimeter does not affect the heat capacity results, in agreement with Sturtevant [17], although the measured heat capacity will correspond to a temperature slightly different from the observed temperature. When the zero heat leak is observed to change between the initial and final equilibration periods the temperature measurement errors do not cancel exactly.

It appears possible to realize a net gain in the precision and perhaps in the accuracy of temperature measurement if the shield control is offset to make the observed temperature constant and the heat which enters the calorimeter at one part of the surface is by-passed around the sample and thermometer, as suggested in the next section, to that part of the surface where it leaves the calorimeter. Such construction might also reduce the possibility that a redistribution of the phases during protracted purity determinations might alter the temperature of the thermometer region relative to the temperature of the interface between the phases.

### 6.2. Correction for the Unknown Heat Exchange

In the usual good practice, the heat capacity of a sample is obtained as the difference between the observed heat capacities of the empty and the full calorimeter. This method allows not only for the heat capacities of the various regions of the calorimeter, but also for the unknown heat exchange, insofar as it is the same in the two experiments. The temperature distribution in the regions of the calorimeter heater and the sample and those regions between and adjacent to them will be especially altered, because the heater supplies much more heat for the same rate of temperature change in the experiments with the full calorimeter than it does in the experiment with the empty calorimeter. If any of these regions is part of the calorimeter surface, data for the empty calorimeter cannot provide an exact correction. In general, loading the sample directly against a thin outer calorimeter wall will alter the unknown heat exchange.

It has been shown above that the transients, which are different for the full and empty calorimeter, nevertheless do not contribute to the unknown heat exchange. The data on the empty calorimeter would therefore make an exact correction for the unknown heat exchange if only the *θ_ic_* on the surface of the calorimeter could be made independent of the *θ_ic_* for the sample and heater, assuming the *θ_is_* are the same for experiments with the empty and full calorimeter.

It is apparent from condition IV–B, table 1, that for heat transfer by conduction only, the *θ_i_* depend on the geometrical distribution of the *θ_j_* and *∂θ_j_/∂n_i_*. If there is interposed, between the surface of the calorimeter and the sample plus heater, a *j*th region in which the geometrical functions *θ_j_* and *∂θ_j_/∂n_i_* are not affected by the sample and heater, then, other things being equal, the *θ_i_* on the surface will be identical in the measurements on the full and empty calorimeter. A *j*th region made of a perfect conductor satisfies the requirements because all derivatives of *θ_i_* are zero and *θ_j_* is constant over the region. Another way to make the surface independent of the sample and heater is to interpose a *j*th region which has nearly a point contact with the *i*th region. The geometric temperature distribution is then exactly defined. Of course, the control temperature must be sensed in or outside this *j*th region or *θ_conc_* will be affected by the heater and sample.

Since the error we seek to avoid is small, it may be possible to approximate closely enough one or both of these arrangements. Figure 1 is a sketch of such a design. The contact regions are small and of a good conductor. The sample region is connected to the calorimeter surface only through the contact region. If direct heat flow from the sample region to the surface (e.g., by radiation) is significant, another thin metallic surface may be interposed to carry this heat back to the contact region. The similar arrangement on the adiabatic shield serves to make the inner metal surface of the shield independent of variations in the environment.

The control temperature is shown measured at the calorimeter contact region. Compared to a thermometer or thermopile distributed over the surfaces, this arrangement would give a quicker response and perhaps make control easier or better. It is also subject to a larger error if the heat transfer coefficient changes between measurements with the empty and full calorimeter. The integral effect of the heat transfer coefficient can be checked experimentally by offsetting the shield control at equilibrium.

Calorimeters have been reported [12, 18] which approximate such a design. The calorimeter surface is made of thin metal insulated by shielding or evacuation from the sample and heater regions except for a limited region of direct metallic contact. In one of these calorimeters, the observed heat of vaporization was not affected by the amount of fluid in the calorimeter; that is, the heat leak from the outer wall was independent of the liquid level and was properly accounted for in the heat leak correction based on data obtained at equilibrium.

To test the efficacy of such a design, the thermal characteristics of the sample region may be changed by changing the amount of sample, its distribution in the calorimeter, or its effective thermal conductivity. Agreement of the data for heat capacity would be a test of the independence of the temperature of the surface from that of the sample and heater. The value of the test would depend on how greatly one could exaggerate the effects of loading. Loading the calorimeter in such a way as to produce the maximum asymmetry should provide a rather severe test. Possibly comparing compact with loose samples or evacuated with helium-filled samples might provide a good test where gas adsorption is not a problem. Changing the effective thermal conductivity of the sample by substituting a fine powder for large crystals may not provide a good test. Small differences in observed heat capacity of fine and coarse samples have been ascribed to surface effects [19]. Reproducibility of the data is a necessary condition to show that the surface temperature distribution is reproduced, but it does not rule out the possibility of compensating effects.

### 6.3. Variation in Heating Rate as a Test

One of the variables which a calorimetrist has at his control is the heating rate of the calorimeter, It seems obvious from the experimental point of view to see if a different heating rate will give different results, and this test has been used by numerous experimentalists [1–11, 22]. For calorimeters which have the properties described in this paper, the observed heat capacity, corrected for the zero heat leak, is independent of the heating rate (sec. 3.2.).

The possibility remains of using the variation in heating rate to test the assumptions made regarding the environment temperature, the shield control, the zero heat leak, the thermal properties, and the decay of transients. Even if a relation can be shown between the heating rate and the first three of these, direct observation appears to be a more sensitive and useful method. It is the author’s opinion that few cases will be found in which the effects of the temperature variation in thermal properties will be revealed by varying the heating rate. The amplitude of transients is proportional to the heating rate, so that variation of the rate should give information about transients. It may be questioned whether this technique will discover transients that are not observed in the equilibration periods.

In continuous heating calorimetry, varying the heating rate can be used to establish the time at which transient effects become negligible. The time response of the system is fixed, but the amplitude of the transients (*τ* and τ*) is proportional to the heating rate. If two experiments are started from the same “rest” temperature but one at double the usual rate, the effect of the transients will be doubled. By observing the time at which the two rates give the same results, one can select a safe “warm-up” period. In intermittent calorimetry, of course, the transients are observed directly in the equilibration periods.

## 7. Conclusions

The treatment of adiabatic calorimeters in this paper assumes that the environment provides a constant-temperature surface, that heat capacities, thermal conductivities, and heat transfer coefficients are independent of temperature over the temperature rise of the experiment, that deviations of the shield control are negligible, that the calorimeter power is constant during the heating period, that the zero heat leak is constant and that transients have become negligible when the final temperature is observed. It is possible to check by observation or by calculation from observations all of these quantities. The approximation of temperature-independent thermal properties, which makes the partial differential equations linear, is implied in nearly all calorimetry, in which the heat observed is corrected for heat exchange during the heating period on the basis of observations during fore and after periods.

Under these conditions, the heat flow equations can be reduced to a sum of solutions satisfying the various boundary conditions and interpretable in terms of tangible physical quantities. In this way, it is possible to analyze the causes and effects of gradients set up by the calorimeter and shield heaters. The heat exchange between the calorimeter and the shield due to the transients at the beginning and end of the experiment cancel for each experiment.

The unknown heat transfer due to the temperature distribution set up during the heating period cannot be detected by varying the heating rate, but with proper precautions in the design, data for the empty calorimeter can be used to make a very good correction for it. The validity of the correction depends on the reproducibility of the gradients between experiments with the full and empty calorimeter. This reproducibility affects the accuracy of the measurements; variations produce a systematic error which cannot be determined experimentally, although experiments with varying sample size and distribution may be used to indicate that the error is small.

In intermittent heating, the time required for the transients to decay is best observed in an equilibration period following heating at the maximum rate possible, which results in the maximum amplitude of the transients. For experiments in which the transient contribution to the temperature measurements is negligible it is valid to use data for the empty calorimeter taken at one rate to correct data for the full calorimeter taken at another rate. Within the limitations imposed by uncertainties in the zero heat leak, the rate of heating can therefore be selected to suit the observer’s convenience in making the power and temperature measurements or maintaining temperature control. This flexibility is especially useful when the sample undergoes a change of state.

Since the zero heat leak depends on the locations of the control temperature sensing elements, an arbitrary offset of the controls to make this heat leak zero introduces no error in the calorimetry. This technique may be used to facilitate calculations, temperature measurement, or observations of protracted processes in the calorimeter.

To permit more thorough study by readers, reports on calorimeters intended for the most careful work might well include, in addition to measurements on Calorimetry Conference samples [20, 21], data taken over that part of the temperature range where the heat transfer coefficient is large on (1) the zero heat leaks and their variation with time, temperature, and the heating rate of the preceding heating period, (2) the overall coefficient for heat transfer between the calorimeter and the shield determined for both the full and empty calorimeter, and (3) the effect of changing the controlled temperature difference between the shield and the environment.

## Figures and Tables

**Table 1:** Equations for heat flow in an adiabatic calorimeter

I	II Temperature distribution (Sum of cols. Ill, IV, and V)	III Temperature distribution due to zero heat leak	IV Temperature distribution due to constant heating	V Transient temperature distribution
				
A. Heat conduction equations	$\begin{array}{l} (1)\;\frac{K_{ic}}{C_{ic}}\nabla^{2}T_{ic}=\frac{\partial T_{ic}}{\partial t}-\frac{\delta_{iw}P_{wc}}{C_{wc}V_{wc}}\\ (2)\;\frac{K_{is}}{C_{is}}\nabla^{2}T_{is}=\frac{\partial T_{is}}{\partial t}-\frac{\delta_{iw}P_{s}}{C_{ws}V_{ws}}\\ \;P_{s}=\int C_{is}\frac{\partial T_{is}}{\partial t}dV+\int_{A_{s}}K_{is}\frac{\partial T_{is}}{\partial n_{is}}dA \end{array}$	$\begin{array}{c} \frac{K_{ic}}{C_{ic}}\nabla^{2}L_{ic}=\beta_{L}\\ \frac{K_{is}}{C_{is}}\nabla^{2}L_{is}=\beta_{L}-\frac{\delta_{iw}P_{L}}{C_{ws}V_{ws}}\\ P_{L}=\beta_{L}\sum_{i}C_{is}V_{is}+\int_{A_{s}}K_{is}\frac{\partial L_{is}}{\partial n_{is}}dA \end{array}$	$\begin{array}{c} \frac{K_{ic}}{C_{ic}}\nabla^{2}\theta_{ic}=\beta_{\theta }-\frac{\delta_{iw}P_{wc}}{C_{wc}V_{wc}}\\ \frac{K_{is}}{C_{is}}\nabla^{2}\theta_{is}=\beta_{\theta }-\frac{\delta_{iw}P_{\theta }}{C_{ws}V_{ws}}\\ P_{\theta }=\beta_{\theta }\sum_{i}C_{is}V_{is}+\int_{A_{s}}K_{is}\frac{\partial \theta_{is}}{\partial n_{is}}dA \end{array}$	$\begin{array}{c} \frac{K_{ic}}{C_{ic}}\nabla^{2}\tau_{ic}=\frac{\partial \tau_{ic}}{\partial t}\\ \frac{K_{is}}{C_{is}}\nabla^{2}\tau_{is}=\frac{\partial (\tau_{is}+\tau_{conc})}{\partial t}-\frac{\delta_{iw}P_{\tau }}{C_{ws}V_{ws}}\\ P_{\tau }=\int_{V_{s}}C_{is}\frac{\partial (\tau_{is}+\tau_{conc})}{\partial t}dV+\int_{A_{s}}K_{is}\frac{\partial \tau_{is}}{\partial n_{is}}dA \end{array}$
B. General boundary conditions.	$K_{i}\frac{\partial T_{i}}{\partial n_{i}}=K_{j}\frac{\partial T_{j}}{\partial n_{i}}+\int_{A}h_{ik}(T_{k}-T_{i})dA_{k}$ *T_i_* = *T_j_*	$K_{i}\frac{\partial L_{i}}{\partial n_{i}}=K_{j}\frac{\partial L_{j}}{\partial n_{i}}+\int_{A}h_{ik}(L_{k}-L_{i})dA_{k}$ *L_i_* = *L_j_* (see note)	$K_{i}\frac{\partial \theta_{i}}{\partial n_{i}}=K_{j}\frac{\partial \theta_{j}}{\partial n_{i}}+\int_{A}h_{jk}(\theta_{k}-\theta_{i})dA_{k}$ *θ_i_* = *θ_j_* (see note)	$K_{i}\frac{\partial \tau_{i}}{\partial n_{i}}=K_{j}\frac{\partial \tau_{j}}{\partial n_{i}}+\int_{A}h_{ik}(\tau_{k}-\tau_{i})dA_{k}$ *τ_i_* = *τ_j_* (see note)
C. Temperature distribution, *t* = 0.	*T_i_=L_i_+T*_0_	*L_i_* independent of time	*θ_i_*=0, *t*<0 and *t* >*t_f_* *θ_i_* are functions of space variables only 0 *<t<t_f_*	$\begin{array}{l} \tau_{ic}=-\theta_{ic}\\ \tau_{is}=-\theta_{is}-\theta_{conc} \end{array}\}\text{ON transient}$
D. Temperature distribution, *t=t_f_*	*T_i_=L_i_*+*T*_0_+*θ*_i_+(*β_L_*+*β_h_*)*t_f_*+*τ*_∞_			$\begin{array}{l} \tau_{ic}^{*}=\theta_{ic}\\ \tau_{is}^{*}=\theta_{is}+\theta_{conc} \end{array}\}\text{OFF transient}$

Note: On boundaries between the calorimeter and the shield and between the shield and the environment the control temperature does not cancel in equations III–B, IV–B, and V–B and must be added to the shield temperature; e.g., *h_ik_*(*θ_ks_*+*θ_conc_*−*θ_ie_*) and *L_ic_ = L_js_*+*L_conc_*. See for example eq (3). For heat leak by radiation from the shield to the environment, *T*_0_ and *T*_0_*_e_* do not cancel. See eq (5).

## Footnotes

1 Figures in brackets indicate the literature references at the end of this paper.

2 The application of the radiation law to this particular case apparently has not been set down explicitly. We assume that absorptivities, emissivities, and the mean temperature do not change significantly during the experiment and that the regions in the apparatus where radiation is important are completely enclosed by surfaces of the calorimeter, the adiabatic shield, or the environment. A fraction *f*_12_ (proportional to *dA*_2_) of the radiation emitted by a differential surface *dA*_1_ strikes *dA*_2_, some by a direct path and some by reflection from other surfaces enclosing the space. That which goes by an indirect path is reflected by the other surfaces dependent on their absorptivities but not on their temperatures. Similarly, a fraction *f*_21_ emitted by *dA*_2_ strikes *dA*_1_ and the net heat transfer by radiation *P*_12_ from *dA*_1_ to *dA*_2_ is given by the equation

$$P_{12}=\alpha_{21}\epsilon_{1}f_{12}\sigma T_{1}^{4}dA_{1}-\alpha_{12}\epsilon_{2}f_{21}\sigma T_{2}^{4}dA_{2},$$

where *ϵ* is the emissivity and *α*_21_ is the absorptivity of surface *dA*_2_ for radiation from surface *dA*_1_, allowing for the fact that most of this radiation is reflected one or more times before striking *dA*_2_ and arrives at various angles. The equation must hold at all temperatures, and, when *T*_1_ = *T*_2_, *P*_12_ must be equal to zero from the second law of thermodynamics. The coefficients of the temperature must therefore be equal and the net radiation may be written

$$P_{12}=\sigma \alpha_{12}\epsilon_{2}f_{21}\left(T_{1}^{4}-T_{2}^{4}\right)dA_{2}.$$

Using the approximation for small *T*_1_ *− T*_2_ [14], one obtains

$$P_{12}=\sigma \alpha_{12}\epsilon_{2}f_{12}T_{m}^{3}(T_{1}-T_{2})dA_{2}\equiv h_{12}(T_{1}-T_{2})dA_{2}.$$

This equation has the same form as Newton’s law of cooling, but it applies to differential areas and radiation only.

3 The assignment of leads, supports, and gas to the shield is arbitrary, but convenient. Their inclusion with the environment would require discussion of environment temperature distributions as well as an environment surface at constant temperature. In a sense, we are evading the question of where the boundaries of the calorimeter and shield are located. For example, a lead wire between the shield and the calorimeter will usually conduct heat away from both of them during the heating period to some unknown part of the wire where the flow from both sides becomes zero. If the zero heat leak is negligible, heat flows out from the calorimeter to this surface, and the heat capacity out to this point is logically part of the calorimeter. If radiation could be neglected, the boundary might be taken as the mathematical surface where *∂θ_ic_*/*∂n_ic_*=0. Then the unknown heat loss is zero and the problem in calibration is to measure the heat capacity within this “surface”. The accuracy of the calorimetry then depends on having the same “surface” in measurements with the calorimeter full and empty. The problem is discussed by Dickenson for a different type of calorimeter [23, sec. III–7].
